# Finite element analysis of a novel repair device for lumbar spondylolysis

**DOI:** 10.1186/s12891-026-10238-9

**Published:** 2026-08-03

**Authors:** Bin Xie, Hongda Xu, Haitao Deng, Mingfan Li, Shengxin Zhao, Youpeng Hu, Li Liu, Peidong Qing

**Affiliations:** 1 Department of Spine Surgery, Mianyang Orthopedic Hospital, Mianyang, Sichuan Province China; 2 Department of Preventive Medicine, Sichuan College of Traditional Chinese Medicine, Mianyang, Sichuan Province China

**Keywords:** Lumbar spondylolysis, Internal fixation, New repair device, Finite element analysis

## Abstract

**Background:**

This study aimed to compare the biomechanical differences between a novel repair device for lumbar spondylolysis and a nail–hook internal fixation system through three-dimensional finite element analysis.

**Methods:**

Three-dimensional images of L4–S1 were constructed based on computed tomography images of a young male patient. Four finite element models were developed: Model A: intact model; Model B: bilateral spondylolysis of the L5 model; Model C: spondylolysis fixed by the nail–hook internal fixation system model; and Model D: spondylolysis fixed by the novel repair device model. A uniformly distributed 600-N vertical downward concentrated force and 10 Nm of torque were applied to the upper surface of the L4 to simulate six physiological activities of the lumbar spine. The fixation effects were evaluated by comparing the range of motion, maximum stress of the intervertebral disks, maximum displacement of the spondylolysis, and stress distribution of the internal fixation.

**Results:**

The range of motions of the two internal fixation models were similar in each segment and direction. In terms of the maximum displacement of the spondylolysis, that of the fracture surface in the novel repair device model was significantly reduced compared with the nail–hook internal fixation model in each range of motion. The maximum stress of the intervertebral disks in the two models had their respective advantages in different activity directions, but the difference in stress values was not significant. The stress levels of both internal fixation devices were concentrated on the connecting rod and the junction of the pedicle screw and the connecting rod. Due to the shorter connecting rod of the nail–hook internal fixation system, the stress on the connecting rod was more concentrated, while the stress distribution on the connecting rod of the novel device was more dispersed.

**Conclusion:**

The new type of lumbar pars interarticularis repair device is highly reliable in fixation, and the bone grafting area of the pars interarticularis is well exposed, providing favorable conditions for bone grafting and subsequent bone formation. It is a highly promising internal fixation device that offers a new option for pars interarticularis repair.

## Introduction

**Fig. 1:**
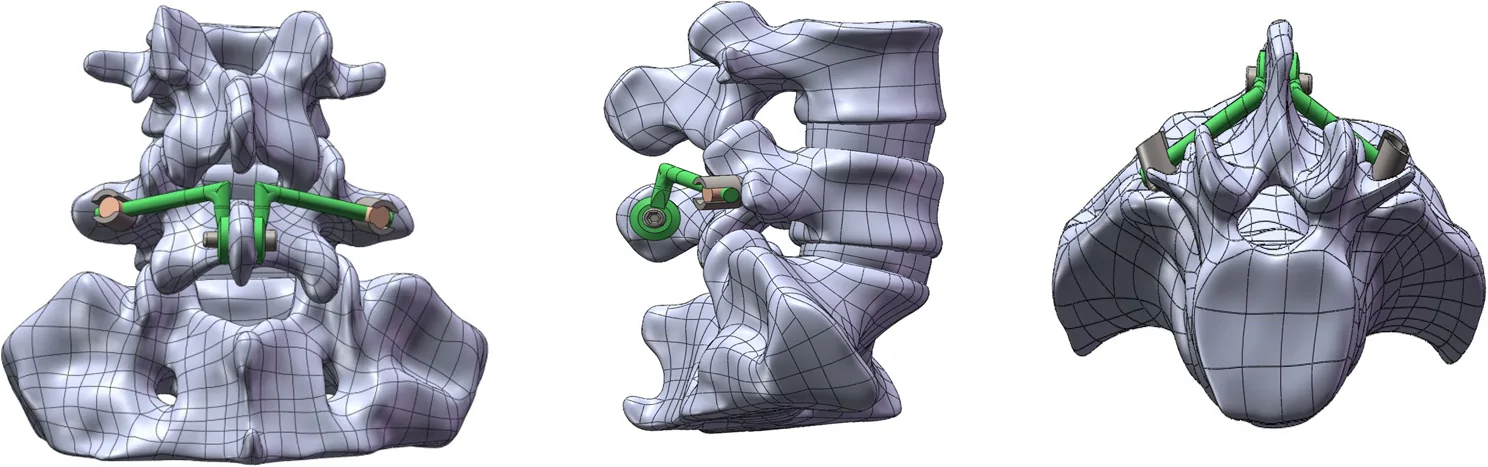
Schematic diagram of the new type of lumbar pars interarticularis repair device

A lumbar pars interarticularis defect refers to a bone defect in the middle part between the superior and inferior articular processes of the lumbar vertebrae [1]. Lumbar spondylolysis, which leads to lumbar instability or even spondylolisthesis, can cause recurrent lower back pain and is a common cause of lower back pain in adolescents. The preferred treatment approach is conservative therapy. If the effect of standardized conservative treatment is poor after 3–6 months and the condition gradually worsens, surgical intervention should be performed. Moreover, for patients who need to engage in physical training over a long period, surgical treatment is more likely to be chosen [2]. Surgical procedures are divided into direct fixation of the isthmic defect within the segment and intersegmental fusion and fixation techniques. The majority of scholars believe that in cases of lumbar spondylolysis without severe slippage, intersegmental fixation and fusion surgery would sacrifice lumbar mobility and no longer meet current medical requirements [3]. Therefore, a large number of scholars more highly recommend the direct fixation of the isthmic cleft within the segment proposed by Buck [4]. Nail–hook internal fixation is currently a relatively common surgical method for repairing the isthmus fissure [5], although it has some drawbacks. The connecting rods of this method pass through the isthmus area, which significantly blocks the bone graft window, resulting in a relatively low fusion rate at the isthmus bone graft site in the later stage [6]. Therefore, our team designed a new type of lumbar pars interarticularis repair device (Fig. 1). While ensuring sufficient strength for fixation, it can expose the bone graft window of the lumbar pars interarticularis as much as possible, ensuring adequate bone grafting and providing favorable conditions for later lumbar pars interarticularis fusion. To verify the effectiveness of this device, we performed three-dimensional modeling and finite element analysis, comparing the novel device with the commonly used nail–hook internal fixation system in clinical practice to evaluate its internal fixation effect and provide a basis for clinical application.

## Materials and methods

### Establishment of the finite element model

This study was based on the original lumbar CT data of a 21-year-old male patient. The slice thickness was 1 mm(This study has been approved by the Ethics Committee of Mianyang Orthopedic Hospital and has received informed consent from the patients themselves). The CT tomographic images were imported into Mimics 21.0 (Materialise, Belgium) to establish an accurate three-dimensional geometric model of the L4–S1 vertebrae and the sacrum. The output STL files were then imported into Geomagic 2018 (Geomagic, USA) for surface configuration and smoothing. Meshing and the assignment of certain material properties were carried out using HyperMesh 2019 (Altair, USA) to obtain a finite element model of the L4–5 segments and the sacrum, including cortical and cancellous bone of the vertebrae, endplates, posterior structures, nucleus pulposus, annulus fibrosus matrix and fibers, and seven related ligaments (anterior longitudinal, posterior longitudinal, ligamentum flavum, joint capsule, interspinous, supraspinous, and transverse ligaments; see Table 1). The cortical bone of the vertebrae forms the outer layer of the vertebrae, with a layer thickness of 1 mm, and each vertebra has an endplate layer approximately 0.8 mm above and below, with cancellous bone inside. The thickness of each intervertebral disk varies, and the anterior and posterior thicknesses of each intervertebral disk also differ, mainly depending on the structure of the scanned vertebrae. The nucleus pulposus of the intervertebral disk occupies approximately 40% of the cross-sectional area of the intervertebral disk at the center. The other external structures of the intervertebral disk are the intervertebral disk matrix, which is composed of six consecutive and intersecting concentric layers of fibrous ring fibers, with each fiber forming an angle of ± 40° with the adjacent endplate. The fibers and ligaments were assumed to be linear elastic materials that bear only tension [7]. The corresponding meshed model file was exported to Abaqus 2019 (Dassault Systèmes France), in which the material properties, boundary conditions, contact, analysis steps, and load row operation analyses were set.

In this study, cortical bone, cancellous bone, and articular endplates were modeled as isotropic and homogeneous linear elastic materials. Each vertebral body consisted of a cortical bone shell surrounding cancellous bone, with a layer thickness of 1 mm(Simplified processing). The upper and lower surfaces of the vertebral body were constructed with endplate shells 0.8 mm thick. The contact properties of the small joint surfaces are set as follows: the tangential action has no friction; the screw and the bone are in a bound contact; the screw and the rod/connection piece are also in a bound contact. The normal action is the hard friction of the “penalty” function. The element types and material properties of each component in the lumbar spine model are shown in Table 1 [8]. 

**Table 1:** Element types and material properties of each component of the lumbar spine model

Spinal structure	element type	Elastic modulus / MPa	Poisson’s ratio	section surface/mm^2^
cortical bone	6-node solid element	12,000	0.30	-
cancellous bone	6-node solid element	150	0.30	-
Rear structure	6-node solid element	3500	0.30	-
Vertebral endplate	6-node solid element	100	0.40	-
nucleus pulposus	8-node solid element	1	0.49	-
Fibrous ring matrix	8-node solid element	4	0.45	-
Fibrous ring fibers	Three-fiber unit	400	-	0.76
anterior longitudinal ligament	Three-fiber unit	8	0.35	49.1
posterior longitudinal ligament	Three-fiber unit	10	0.35	22.2
ligamenta intertransversaria	Three-fiber unit	5	0.35	4
Joint capsule ligament	Three-fiber unit	5	0.35	103.9
ligamenta interspinalia	Three-fiber unit	5	0.35	49.2
supraspinal ligament	Three-fiber unit	5	0.35	70.3
ligamentum flavum	Three-fiber unit	5	0.35	71.1
Spinal implants (made of titanium alloy)	6-node solid element	110,000	0.30	-

In this model, the nucleus pulposus region uniformly adopts the C3D8H hybrid composite element to avoid volume locking

### Load and boundary condition settings

**Fig. 2:**
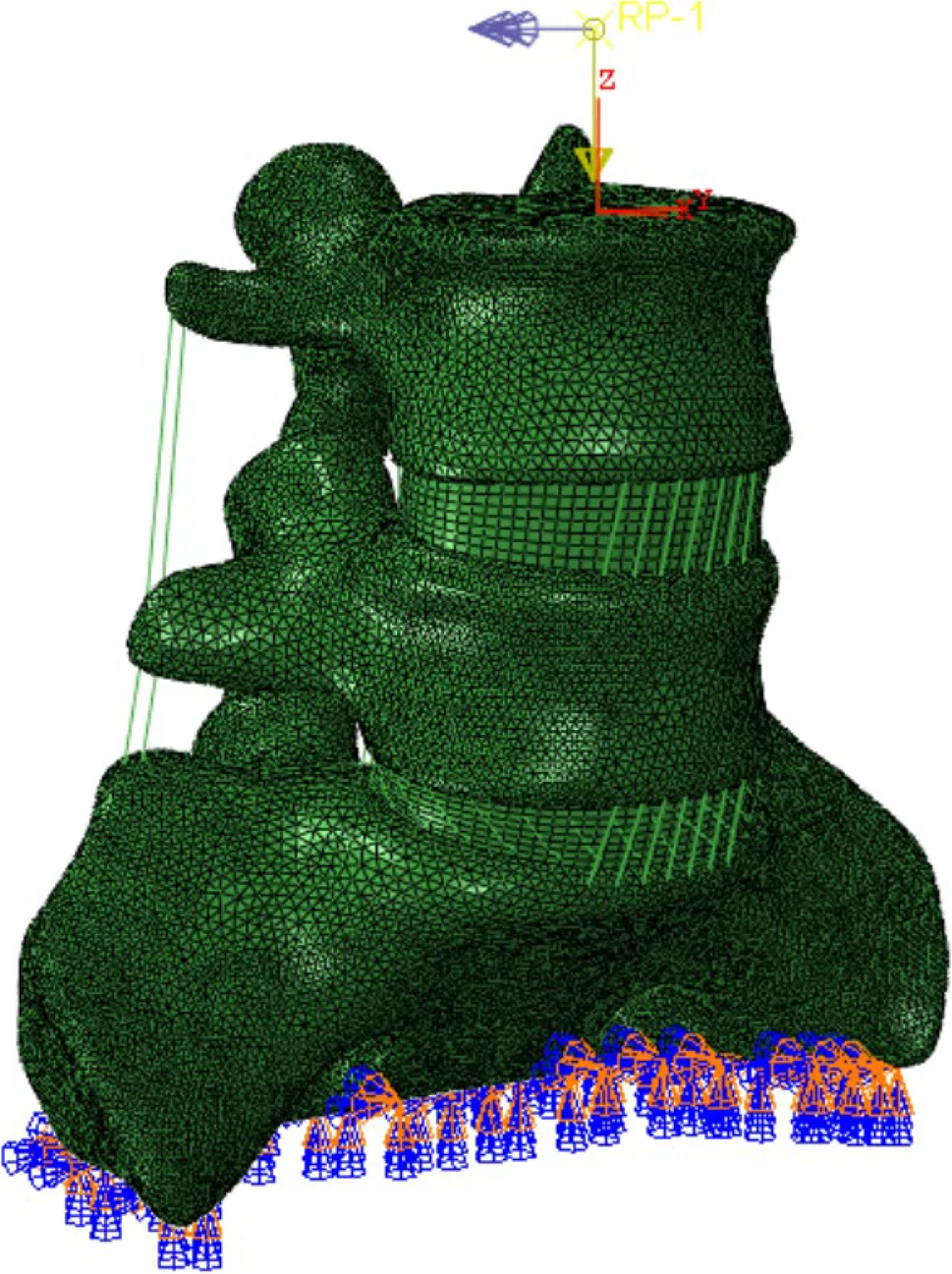
Load and boundary condition settings

Based on the previously measured human biomechanical data. The loading process first applies a preload of 600 N [9], representing the weight of the upper body and the pulling effect of the muscles. The method is to couple the centrum with its center of mass, and connect all the centers of mass to form a loading path. A contraction force is applied along this path. A torque of 10Nm [10] is applied, and the six movement directions of the lumbar spine model are tested. At the same time, all the degrees of freedom at the bottom of S1 need to be constrained. (Fig. 2)

### Model validation

**Fig. 3:**
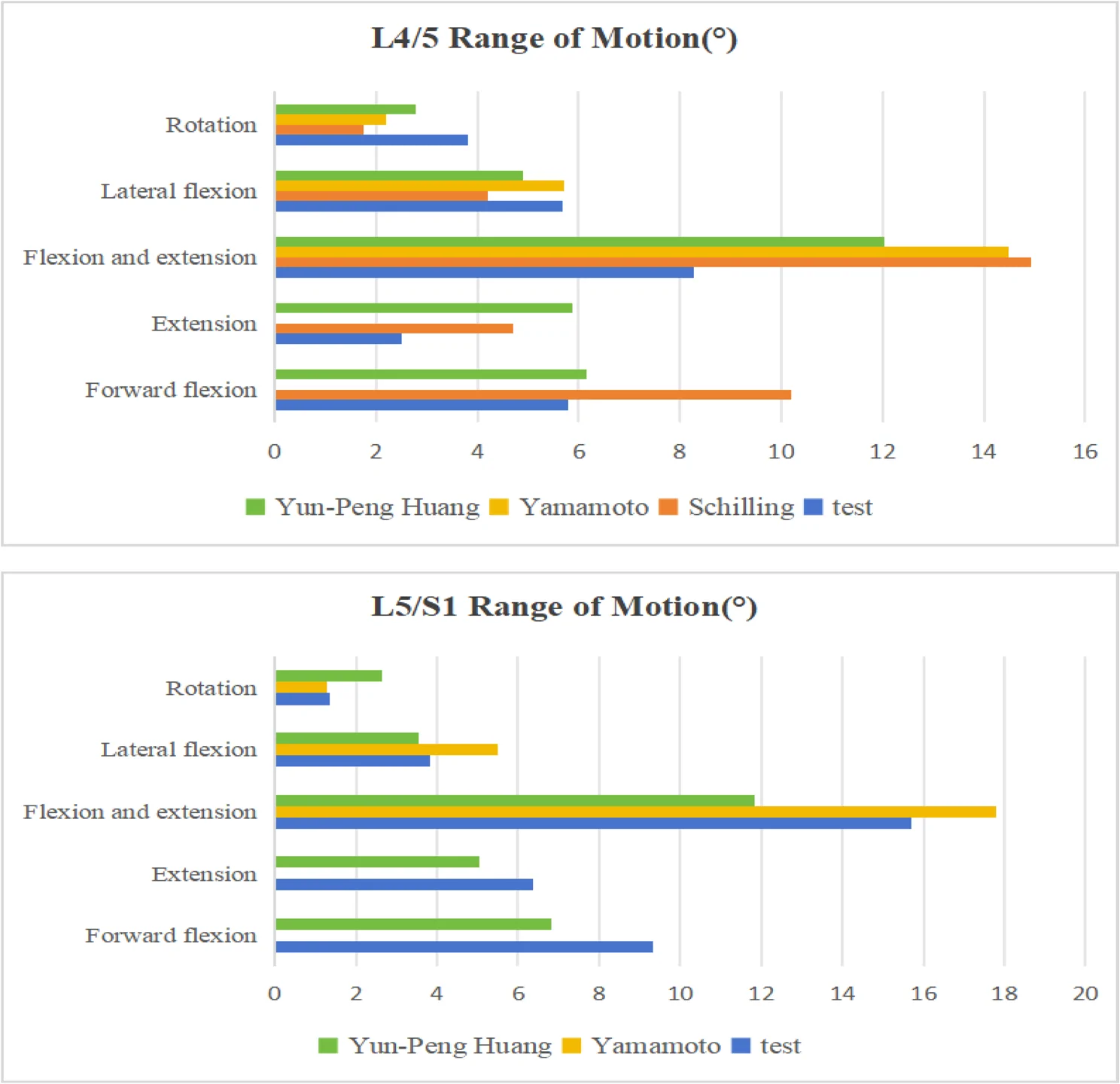
Normal L4-S1 vertebral body model and three-dimensional range of motion (°) of each L4-S1 vertebral segment in in vitro biomechanical experiments and finite element models of other researchers

The overall model constructed using the finite element method was compared with in vitro biomechanical experimental data [11, 12] and the finite element models of other researchers [13]. Differences in lumbar spine mobility were examined to evaluate the validity of the finite element model. (Table 2; Fig. 3)

**Table 2:** Three-dimensional range of motion (°) of each L4-S1 vertebral segment in the normal L4-S1 vertebral body model and in the in vitro biomechanical experiments and the finite element models of other researchers. The percentage error between the current study model and Yamamoto’s in vitro experimental activity ranges was as follows: flexion/extension (L4/5: 2.97%, L5/S1: -11.74%), lateral bending (L4/5: 26.14%, L5/S1: 30.55%), and rotation (L4/5: 20%, L5/S1: 3.08%)

	Complete model		Schilling’s [12] in vitro experiment	Yamamoto’s [11] in vitro experiment		Yun-Peng Huang’s [13] finite element model	
	L4-5	L5-S1	L4-5	L4-5	L5-S1	L4-5	L5-S1
Forward flexion	10.21	9.34	5.78	/	/	6.15	6.81
Extension	4.72	6.37	2.50	/	/	5.88	5.04
Flexion and Extension	14.93	15.71	8.28	14.5	17.8	12.03	11.85
Lateral flexion	4.21	3.82	5.68	5.6	5.5	4.91	3.55
Rotation	1.76	1.34	3.82	2.2	1.3	2.78	2.64

### Construction of the experimental model

**Fig. 4:**
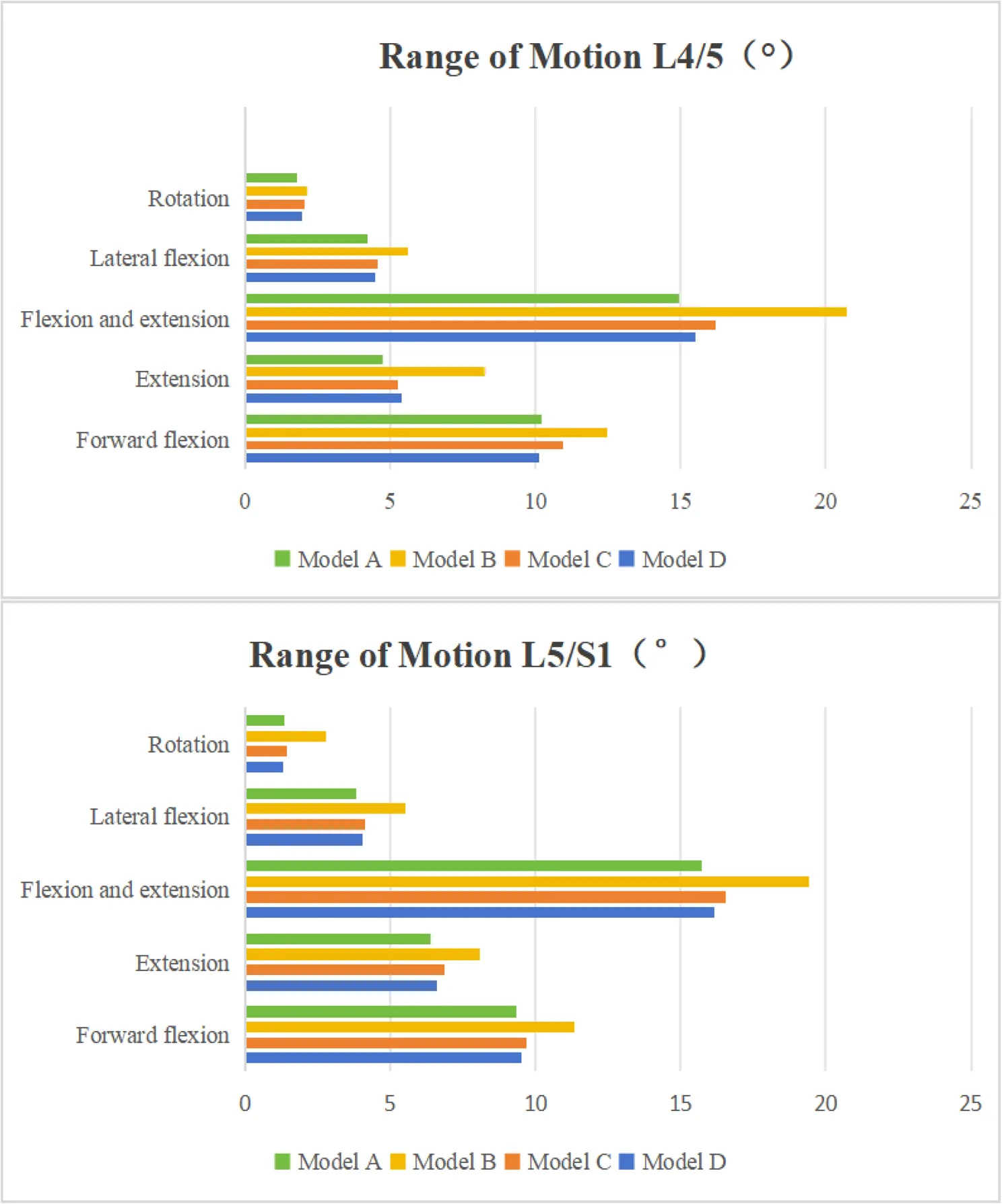
Range of motion of the L4-5 and L5-S1 segments for each model; In the flexion position: The range of motion of L4-5 is B > C > A > D; The range of motion of L51-S1 is B > C > D > A;In the extension position: The range of motion of L4-5 is B > D > C > A; The range of motion of L5-S1 is B > C > D > A;In the flexion-extension position: The range of motion of L4-5 is B > C > D > A; The range of motion of L5-S1 is B > C > D > A;In the left/right bending position: The range of motion of L4-5 is B > C > D > A; The range of motion of L5-S1 is B > C > D > A;In the left/right rotation position: The range of motion of L4-5 is B > C > D > A; The range of motion of L5-S1 is B > C > A > D;

Four finite element models were used in this experiment (Fig. 4): Model A: a complete L4–S1 centrum model; Model B: an L4–S1 centrum model with L5 bilateral isthmic fissures; Model C: Model B with a nail–hook internal fixation device applied; and Model D: Model B with the new repair device applied. A uniformly distributed 600-N vertical downward concentrated force was applied to the upper surface of all models at L4 to bring them close to the physiological state. Then, a 10 Nm torque was applied to simulate the flexion, extension, left–right axial rotation, and left–right lateral bending of the spine. The range of motion (ROM) of the L4–5 and L5–S1 segments, the maximum stress of the intervertebral disks, the maximum displacement of the pedicle, and the stress distribution of the internal fixation were recorded.

## Results

### Model validation

ROMs of flexion, extension, lateral bending, and axial rotation for the L4–5 and L5–S1 segments in the normal finite element model were recorded, and the results were compared with those reported by other researchers in in vitro experiments and finite element models. In this study, the ROM of the L4–S1 lumbar spine obtained using the finite element model was within the standard deviation of the average values of other in vitro experiments and finite element models, and the constructed model was deemed effective. (Table 2; Fig. 3)

### Range of motion

**Fig. 5:**
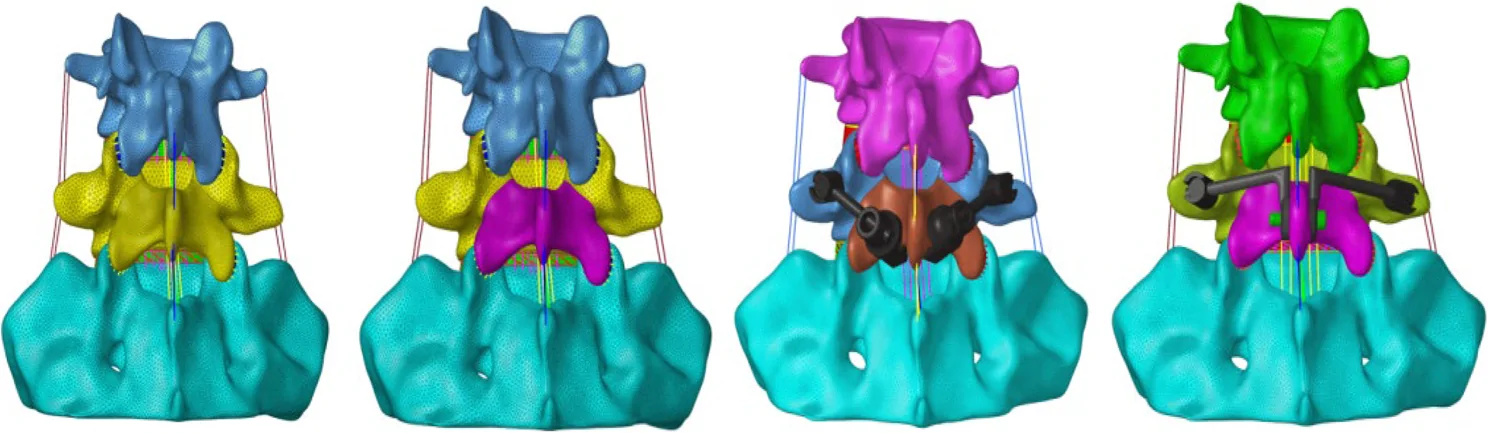
Four finite element models

The ROMs of the L4–5 and L5–S1 segments for the four models are shown in Fig. 4. Among them, the ROMs of each segment in Model B were greater than those in Models A, C, and D. Model C showed increased ROM in all directions compared with Model A, while Model D exhibited increased ROM in all segments and directions except for L4–5 flexion and L5–S1 rotation, in which the ROM was smaller than that of Model A. However, models C and D had similar ROMs for each segment and direction. The finite element model is shown in Fig. 5.

### Maximum model shift

**Fig. 6:**
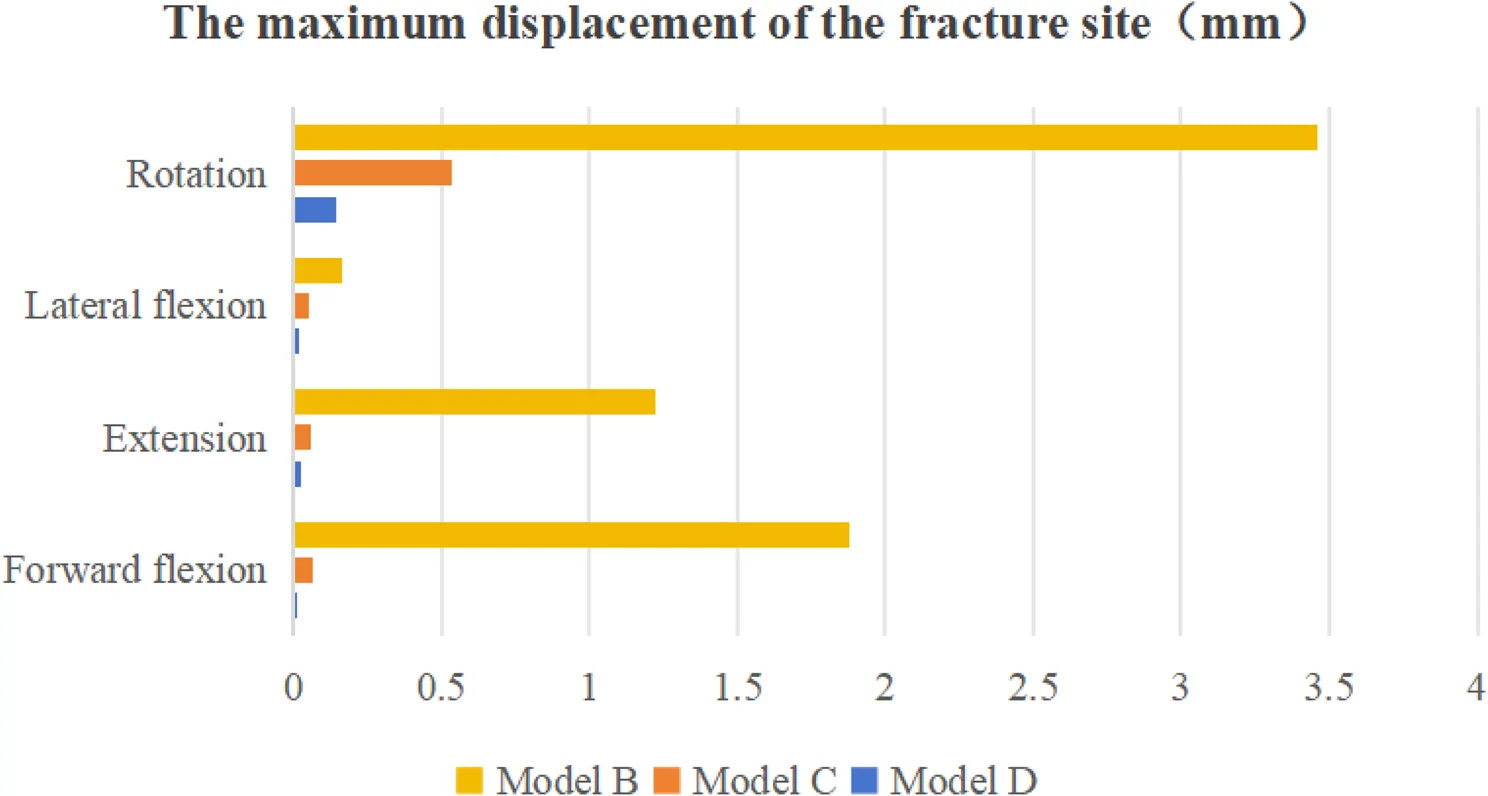
Maximum displacement of the fracture surfaces of the isthmus in models B, C, and D

The maximum displacement on the fracture surface occurs during left/right rotation. By measuring the overall displacement of the model and then measuring the relative displacement of each point on the fracture surface, the displacement can be calculated. The maximum displacement levels of the fracture surface of the isthmus in models B, C, and D are shown in Fig. 6. Compared with Model B, these were significantly reduced in all ROMs (flexion, extension, left/right bending, and left/right rotation) in Models C and D. In Model D, they were significantly lower than those in Model C across all ROMs.

### Stress distribution of intervertebral disks

**Fig. 7:**
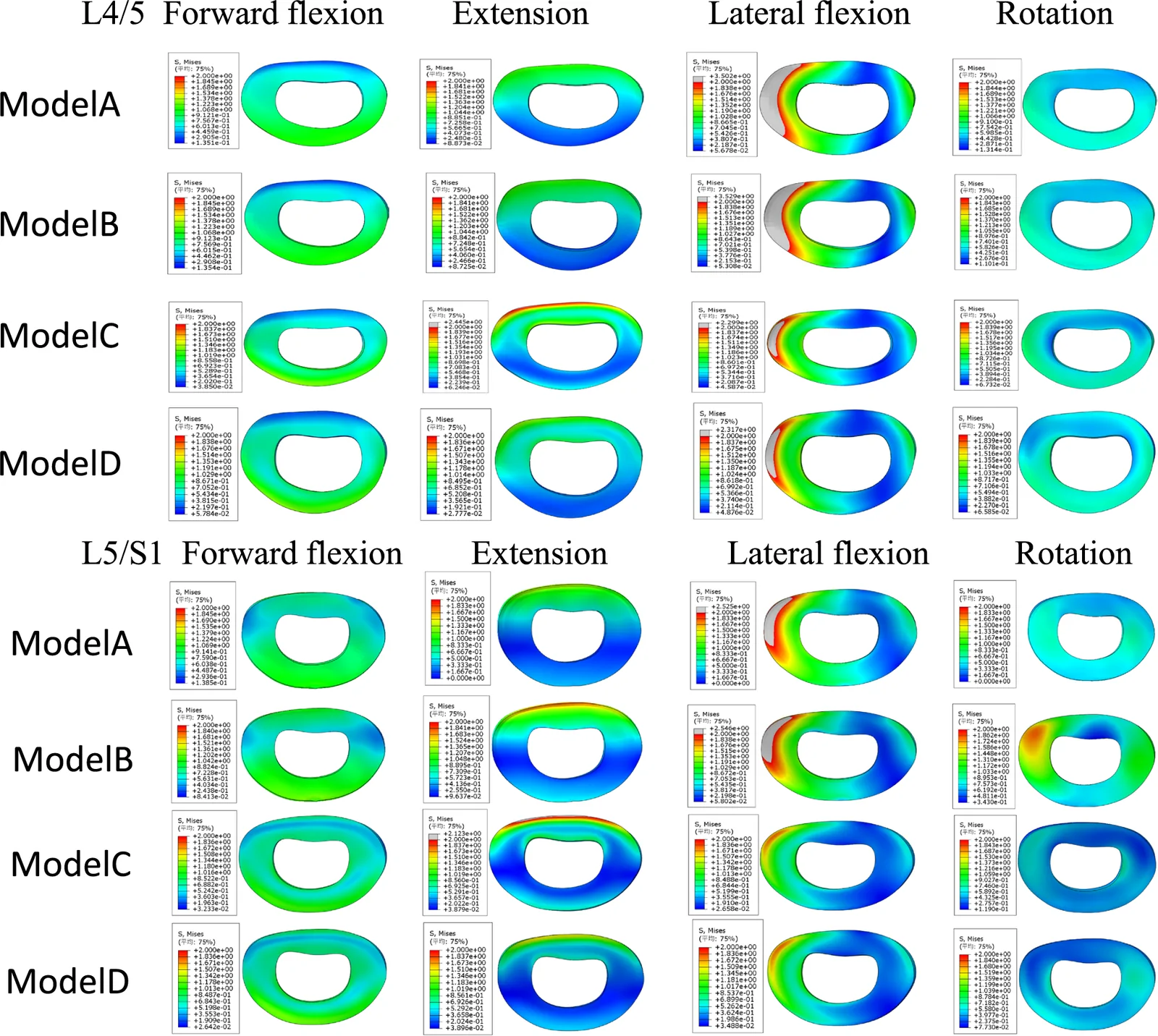
Stress distribution of intervertebral discs in four models

**Fig. 8:**
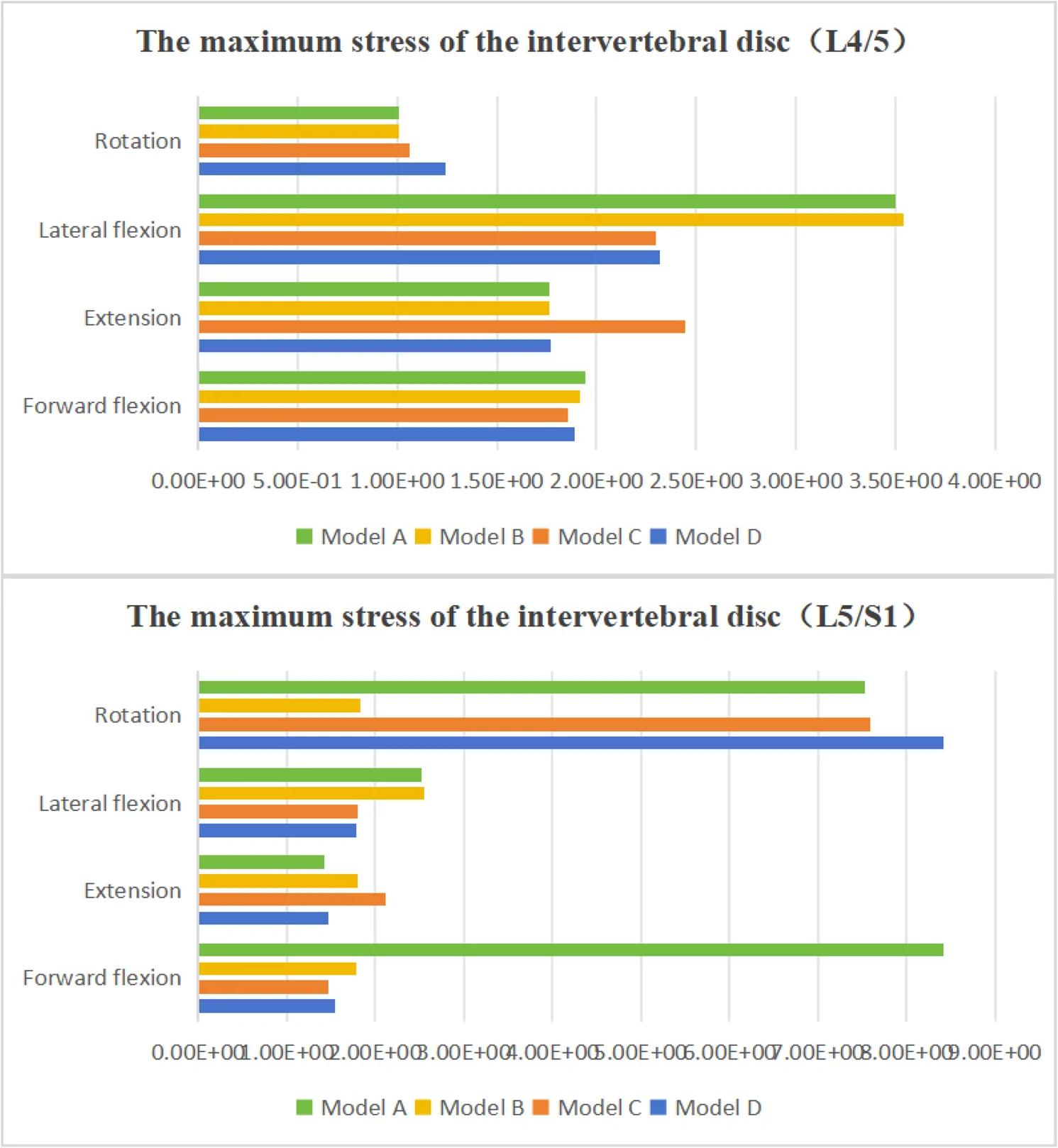
Maximum stress values of intervertebral discs under each ROM for the four models; In the flexion position, the maximum stress of the L4/5 intervertebral disc is A > B > D > C; the maximum stress of the L5/S1 intervertebral disc is B > D > A > C;In the extension position: the maximum stress of the L4/5 intervertebral disc is C > D > A > B; the maximum stress of the L5/S1 intervertebral disc is C > B > D > A;In the left/right bending position: the maximum stress of the L4/5 intervertebral disc is B > A > D > C; the maximum stress of the L5/S1 intervertebral disc is B > A > C > D;In the left/right rotation position: the maximum stress of the L4/5 intervertebral disc is D > C > B > A; the maximum stress of the L5/S1 intervertebral disc is D > C > A > B;

The stress analysis of the intervertebral disks for the four models showed that in the flexion and extension states, the stress at their anterior and posterior edges was more concentrated. In the left and right lateral bending states, the stress distribution was concentrated on the left and right edges of the intervertebral disks, taking left lateral bending as an example. In the left and right axial rotation states, the stress was more concentrated on the anterior and lateral sides of the intervertebral disks. The stress distributions of the intervertebral disks across the four models are shown in Fig. 7, and the maximum stress values under each ROM are shown in Fig. 8.

### Maximum stress and distribution of the internal fixation system

**Fig. 9:**
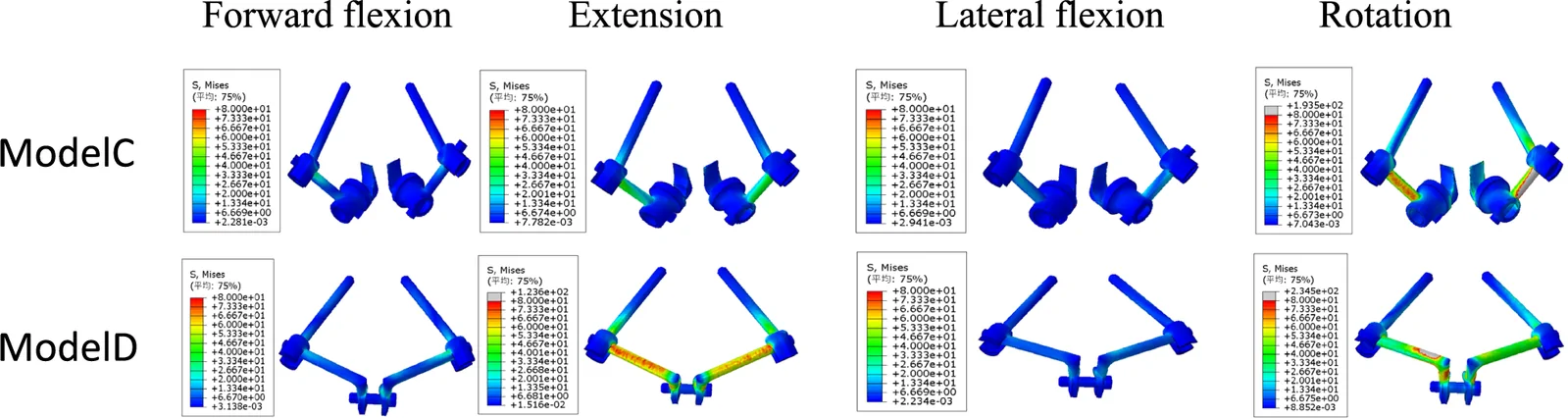
Stress distribution map of internal fixation in models C and D

In Models C and D, the stress was concentrated on the connecting rods and the junction between the connecting rods and the screws in different activity directions. Model D had a more dispersed stress distribution, but the maximum stress value was larger. (The stress distribution is shown in Fig. 9, and the maximum stress value is shown in Table 3.)

**Table 3:** Comparison of the maximum stress values in the internal fixation of Model C and Model D

maximum stress	Model C(Mpa)	Model D(Mpa)
Rotation	4.641e + 1	4.603e + 1
Lateral flexion	7.627e + 1	1.236e + 2
Extension	3.066e + 1	4.011e + 1
Forward flexion	1.935e + 2	2.345e + 2

## Discussion

A lumbar pars interarticularis fissure is a common cause of lower back pain in adolescents [14–16]. It occurs most frequently at the L5 segment, accounting for 97% of cases, and bilateral fissures account for 88%. Among adult cases with pars interarticularis fissure, L5 accounts for 90%, while in adolescents it accounts for 65%–85% [17]. Fujii et al. [18] classified diaphragmatic fissures into three stages based on their CT manifestations: stress response, stress fracture, and diaphragmatic defect (non-healing) stages, corresponding to the early, progressive, and terminal stages, respectively. In some cases in the progressive and late stages that did not receive strict conservative treatment, the bone defects could not heal on their own. If the condition continued to progress, it could develop into lumbar spondylolisthesis. In progressive-stage cases that were ineffective with conservative treatment and in those with bone defects, surgical intervention was necessary.

### Surgical methods for lumbar pars interarticularis fractures

Based on the pathological mechanism of lumbar pars interarticularis fissure and on previous research, the key to surgical repair is to completely remove the scar tissue at the fracture ends of the pars and fill it with bone grafts, combined with strong internal fixation. This ensures the relative stability of the pars while restoring the continuity and integrity of the bone [1, 19, 20]. Therefore, the choice of internal fixation method has become the focus of debate among scholars. The current commonly used surgical methods in clinical practice can be divided into two main categories: segmental direct fixation of the isthmus fissure, proposed by Buck [4] in 1970, and the intervertebral fusion fixation technique using pedicle screw internal fixation systems developed by various scholars [21, 22]. With the deepening of biomechanical research in recent years and the advancement of the concept of accelerated rehabilitation surgery, most scholars believe that in cases of lumbar isthmus fissure without severe slippage, segmental interfusion surgery no longer meets current medical requirements. This is because intervertebral fusion surgery fuses the diseased vertebra with the adjacent vertebrae, resulting in the loss of mobility of the diseased vertebra and sacrificing the normal physiological movement unit of the patient’s lumbar spine. Moreover, it may accelerate the degeneration of the adjacent segmental lumbar intervertebral disks [3].Therefore, scholars are increasingly attaching importance to segmental fixation techniques, including transpedicular puller screw fixation (the Buck method [4]), transverse process–sacral process wire fixation (the Scott method [23]), and nail–hook internal fixation [24]. However, the Buck technique poses the risk of stress concentration, which may cause the internal fixation screws to break, and this surgery requires a limited amount of effective bone grafting [25]. In a finite element analysis of this internal fixation, it was pointed out that under the conditions of posterior extension and trunk rotation, the stress on the isthmus and the screw increased the most, which is a high-risk mechanical action after the surgery [26]. This study also compared the kinematics, bone, intervertebral discs and internal fixation stresses of three different fixation methods: simple Buck segmental screws, simple inter-segmental pedicle screws, and Buck + pedicle screw hybrid fixation. It was concluded that the Buck + pedicle screw hybrid fixation had the best comprehensive biomechanical performance: however, it sacrificed some lumbar spine mobility. The Scott technique requires extensive tissue and iliolumbar ligament resection to expose the transverse process, and it may cause transverse process fractures, resulting in loosening of the steel wire and nonunion of the fracture site, which can cause persistent chronic low back pain in clinical practice [27, 28]. The nail–hook internal fixation covers more of the bone graft window, resulting in a lower posterior fusion rate in the later stages [6]. This method achieves proximity of the defect area of the posterior arch by applying pressure between the plate hook and the pedicle screw. However, most patients with lumbar posterior arch defects have congenital developmental abnormalities, and this process may cause the bony structure of the posterior arch to invade the spinal canal during the compression process [29]. Researchers have proposed novel strategies for the direct repair of posterior arch defects. Huang et al. [30] used a V-titanium cable combined with a pedicle screw fixation system to treat the fifth lumbar posterior arch defect in young people with good results, but unfortunately, their study did not have long-term follow-up outcomes. Berjano [31] introduced the pedicle screw, rod, and polyester band technique, which significantly alleviated patients’ pain and accelerated their social rehabilitation over a 4-year follow-up period. However, this technique requires extensive tissue anatomy and is currently limited to case reports. There have been no further studies with a larger sample size to verify the results.

### Novel repair device for lumbar spondylolysis

Our research team designed a new type of repair device for lumbar pars interarticularis. The aim was to implement the concept of segmental internal fixation while achieving the goal of complementing and improving other fixation methods. This device consists of pedicle screws and L-shaped right-angle rods, which connect the pedicles and spinous processes at both ends of the pars interarticularis. The L-shaped right-angle rods are used to bypass the location of the pars interarticularis, achieving successful treatment of the pars bone window and enabling sufficient bone grafting. During the locking process of the screws on both sides of the spinous processes, the L-shaped right-angle part can adhere closely to the spinous processes, achieving compression fixation. To further verify the effectiveness of this novel repair device, three-dimensional reconstruction and finite element analysis were conducted. The device was also compared with the commonly used nail–hook internal fixation system in clinical practice. Four three-dimensional finite element models were built based on CT data. The models were compared in terms of lumbar spine mobility, maximum displacement of fractures, stress distribution in intervertebral disks, and stress distribution in internal fixation. In terms of lumbar spine mobility, the nail–hook internal fixation model showed increased mobility across all directions compared with the normal model. The novel repair model had less mobility in L4–5 flexion and L5–S1 rotation compared with the current model, except for other segments and directions, in which mobility increased compared with the normal model. The two internal fixation models had similar mobility across all directions. In terms of the maximum displacement of the isthmus fracture, the new repair device model had a smaller maximum displacement on the isthmus fracture surface across all ROMs than the nail–hook model. Generally, we believe that a smaller maximum displacement indicates a more reliable fixation effect [26]. We determined that this might be due to the right-angle compression effect of the connecting rod. Stress analysis of the intervertebral disks of the two internal fixation models showed that the maximum stress followed these patterns: C < D in the anterior flexion state; C > D in the posterior extension state; C < D in the left and right lateral bending states for the L4–5 segment and C > D for the L5–S1 segment; and C < D in the left and right rotation states. Although the maximum intervertebral disk stress in each activity direction of the two internal fixation models had its own advantage, the differences in stress values were not obvious. After observing the stress distribution of the two internal fixation models, we found that for both internal fixation devices, the stress was concentrated at the connection rod and at the junction of the pedicle screw and the connection rod. Because the connection rod of the nail–hook system is shorter, the stress on it was more concentrated, whereas the stress distribution on the connection rod of the new repair device was more dispersed. Some researchers believe that this dispersed stress may produce a smaller shear force in the local area, which is more conducive to early fibrous callus formation and subsequent bone callus formation [32]. This study did not conduct fatigue life analysis or safety factor assessment, so it is still unknown which of the two internal fixation methods has better biomechanical performance. In this study, the screw, the connecting rod, and the bone interface were set as a bound constraint, which was done to reduce the computational complexity and improve the convergence of the solution. This assumption restricts the relative sliding and micro-motion between the interfaces, and there is a difference from the actual mechanical state in clinical and in vitro experiments where there can be slight sliding and loosening at the connection site. This difference may be more pronounced in cases of osteoporosis. As pointed out in a finite element analysis regarding osteoporosis in adolescents with spinal curvature, osteoporosis significantly increases the overall displacement of the lumbar vertebrae, reduces structural stability, and leads to significant asymmetric stress concentration [33].Of course, all the models have the same settings, so the core qualitative conclusions such as the patterns of inter-group horizontal comparison, the trend of stress distribution, and the location of stress concentration remain reliable. The maximum stress of the novel repair device was larger than that of the nail–hook internal fixation system, and the peak was concentrated at the right angle of the connection rod. Since some reports have suggested that the stress size and distribution of internal fixation are related to postoperative internal fixation loosening and fracture [34]. However, the design load of model D is much lower than this. Additionally, the stress distribution within this model is more dispersed, and the overall structure’s cyclic stress level is lower, which is beneficial for enhancing the overall fatigue life. Although there is a peak at the right angle, this peak is still below the material’s fatigue limit, and we have specifically enhanced the fatigue resistance at this location through local reinforcement (The outer diameter dimension has been reinforced from *R* = 4 to *R* = 0.5). In conclusion, we believe that the novel repair device provides strong fixation and can fully expose the bone graft window, thereby creating favorable conditions for later bone formation. It represents a new option for the direct repair of isthmus fissures.

### Limitations

Firstly, our research only verified the reliability of the new repair device at the three-dimensional finite element level, but did not validate the biomechanical properties of the samples. The advantages of finite element analysis lie in its standardized operation and strong comparability of results. It is often used to evaluate the relative biomechanical performance changes of the lumbar spine under different pathological conditions (such as intervertebral disc degeneration, internal fixation devices). However, its greatest limitation is that, in order to achieve standardization and simplification, some biological mechanical authenticity is sacrificed, especially in aspects such as load transmission path, muscle dynamic system simulation, and boundary conditions. From the model validation process of this study, it can also be observed that the finite element model significantly outperforms the classical in vitro experimental data in terms of the range of motion (ROM). This is because the isolated specimens are “rigid mechanical structures”, while the in vivo models are “flexible biological composite structures”, and their mechanical properties inherently differ [35]. The soft tissue has low stiffness during small deformation and its stiffness sharply increases after large deformation. The linear elasticity assumption of this model cannot simulate the subsequent hardening, resulting in the joint being able to undergo continuous large flexion and extension. Insufficient stiffness will cause an increase in structural flexibility. This leads to the equivalent load being absorbed by the deformation, a reduction in the global stress amplitude, but the location and temporal sequence of stress concentration are not significantly affected [36]. The standardized verification system for biomechanical finite element models established by Metar et al. also points out that kinematic accuracy and overall structural stiffness are the core prerequisites for ensuring the reliability of stress calculations and failure analysis [37]. This study is affected by the insufficient overall stiffness of the model and the relatively large range of motion. As a result, the calculated peak stress and the degree of stress concentration are underestimated, which will to some extent weaken the quantitative assessment of the risk of local failure [38]. Meanwhile, this study only conducted static load analysis. Most cases of lumbar pars fractures occur in people with high physical activity levels. Their lumbar vertebrae are repeatedly subjected to force, resulting in cyclic alternating stress, which is prone to cause fatigue damage. Static simulations can only show the stress situation under a certain posture, but cannot reproduce the load changes and fatigue effects during the movement process. In the future, by optimizing the constitutive model of soft tissues and calibrating the material stiffness parameters, the quantitative accuracy of the simulation results will be further improved, and dynamic cyclic load analysis will be added to assess the damage and failure risks of internal fixation under dynamic conditions. Finally, In this study, an anatomical model of the L4-S1 segment for a single healthy young male was constructed, without constructing a model for the entire lumbar spine. All the mechanical analysis results were based on the specific bony morphology, intervertebral disc size, spinal canal geometry, and soft tissue anatomical parameters of this model. There were anatomical differences among individuals, such as vertebral body size, intervertebral disc thickness, ligament attachment position, bony curvature, and muscle tension. Therefore, the quantitative results of this study have certain deviations and cannot be fully generalized to different age, gender, body type, and degeneration degree populations. In the future, a multi-subject, multi-age, and full-lumbar-spine anatomical model will be included to further verify the universality of the mechanical laws.

## Conclusion

The new type of lumbar pars interarticularis repair device is highly reliable in fixation, and the bone grafting area of the pars interarticularis is well exposed, providing favorable conditions for bone grafting and subsequent bone formation. It is a highly promising internal fixation device that offers a new option for pars interarticularis repair.

## Data Availability

The datasets used and/or analyzed during the current study are available from the corresponding author on reasonable request.

## Abbreviations

CT: Computed tomography
L4: Lumbar vertebra 4
L5: Lumbar vertebra 4
S1: S1 vertebra
ROM: Range of motion
Mpa: Megapascal
